# Accuracy of pedicle screw placement in patients with Marfan syndrome

**DOI:** 10.1186/s12891-017-1490-8

**Published:** 2017-03-21

**Authors:** Jun Qiao, Feng Zhu, Leilei Xu, Zhen Liu, Xu Sun, Bangping Qian, Qing Jiang, Zezhang Zhu, Yong Qiu

**Affiliations:** 1 Department of Spine Surgery, the Affiliated Drum Tower Hospital of Nanjing University Medical School, 321 Zhongshan Road, Nanjing, China; 2 Department of Orthopedics, the Affiliated Drum Tower Hospital of Nanjing University Medical School, Nanjing, China

**Keywords:** Pedicle screw, Marfan syndrome, O-arm navigation

## Abstract

**Background:**

There is no study concerning safety and accuracy of pedicle screw placement in Marfan syndrome. The objective of this study is to investigate accuracy and safety of pedicle screw placement in scoliosis associated with Marfan syndrome.

**Methods:**

CT scanning was performed to analyze accuracy of pedicle screw placement. Pedicle perforations were classified as medial, lateral or anterior and categorized to four grades: ≤ 2 mm as Grade 1, 2.1–4.0 mm as Grade 2, 4.1–6.0 mm as Grade 3, ≥6.1 mm as Grade 4. Fully contained screws or with medial wall perforation ≤ 2 mm or with lateral wall perforation ≤ 6 mm and without injury of visceral organs were considered acceptable, otherwise were unacceptable.

**Results:**

976 pedicle screws were placed, 713 screws (73.1%) were fully contained within the cortical boundaries of the pedicle. 924 (94.7%) screws were considered as acceptable, and 52 (5.3%) as unacceptable. The perforation rate was higher using free-hand technique than O-arm navigation technique (30.8% VS. 11.4%, *P* < 0.05), higher in lumbar region than in thoracic region (34.1% VS. 22.3%, *P* < 0.05) and higher in concave side than in convex side (33.5% VS. 21.9%, *P* < 0.05). No injury of visceral organs especially aorta erosion was noted in the series. 7 cases of dural tear caused by misplaced screws occurred, and 4 cases developed cerebro-spinal fluid leak. Drainage and pressure dressings were applied for these patients, and no infection was observed. Leg pain was observed in 7 cases, and 2 cases simultaneously complained of leg weakness. Revision surgery was conducted to remove the misplaced screws for these 2 patients. Conservative treatment was applied for the 5 patients without leg weakness. Symptoms of leg weakness and pain resolved in all patients.

**Conclusion:**

Placement of pedicle screw in Marfan syndrome is accuracy and safe. O-arm navigation was an effective modality to ensure the safety and accuracy of screw placement. Special attention should be paid when screws were placed at the lumber spine and the concave side of spine deformity to avoid the higher rate of complications.

## Background

Marfan syndrome, a disease caused by an autosomal dominant mutation of the fibrillin-l gene occurred in around two to three in 10,000 people [1–3] Many systems including respiratory system, skeleton, cardiovascular system, eyes and skin could be involved [4].

**Fig. 1:**
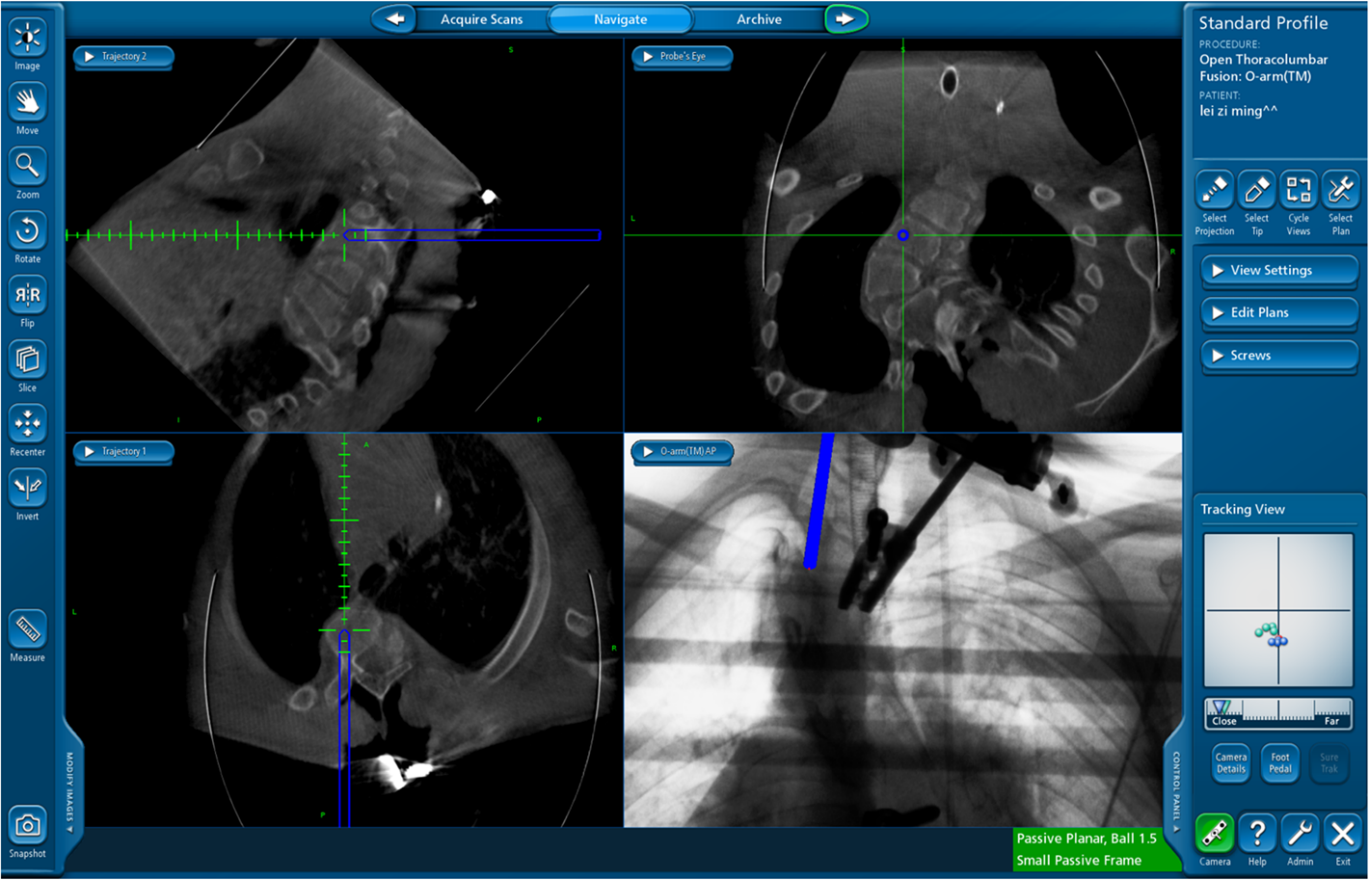
O-arm navigation system demonstrated ideal entry point and trajectory of screws

**Fig. 2:**
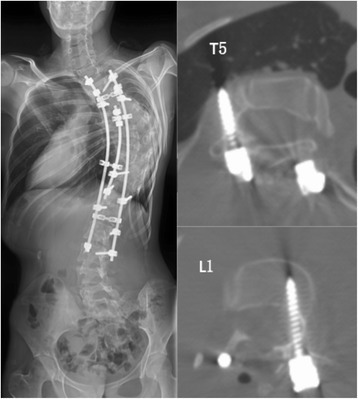
Postoperative CT scanning demonstrating lateral perforation in T5 level and medial perforation in L1 level

**Fig. 3:**
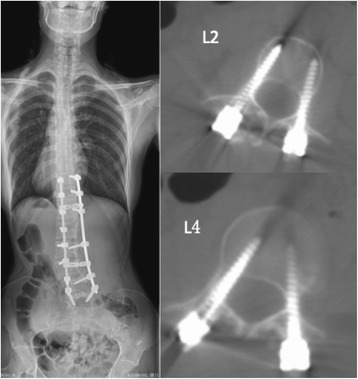
Postoperative CT scanning demonstrating thin pedicle could not contain screw in L2 level and medial perforation in L4 level

The incidence of scoliosis ranges from 52% to 100% in patients with Marfan syndrome [5]. Although the curve pattern of scoliosis with Marfan syndrome is similar to idiopathic scoliosis, the rigidity and natural history is more similar to neuromuscular scoliosis. [6–8]. Brace treatment is used for patients with mild to moderate curves, but always ineffective. The reported successful rate is only 17% for a group of patients with mild to moderate curves [9]. Posterior instrumentation has been largely used to treat the scoliosis in Marfan syndrome [10]. Harrington rods and sublaminar wires were widely used in the past. However, laminar fracture and hook dislodgment occurred more often because of dural ectasia and the thin laminae in Marfan syndrome [11, 12]. Currently pedicle screw fixation is the preferred method of treatment in scoliosis surgery because it offers superior correction [13]. Compared to the hook system, pedicle screw fixation provides stronger corrective forces for fixing three columns simultaneously. The pedicle system is more suitable for the correction of scoliosis in Marfan syndrome because it can purchase on the pedicle and vertebral body even when the lamina is too fragile to be anchored in deformed spine. However, pedicle screw placement carry the potential risks of neurological, vascular or visceral complications including injuries of spinal cord, nerve root and aorta especially in Marfan syndrome featuring dystrophic pedicle and dural ectasia [11, 12, 14, 15]. The safety and accuracy of pedicle screw placement in adolescent idiopathic scoliosis have been well documented, and there is no such study concerning those of the pedicle screw placement in Marfan syndrome. This study is aimed to investigate accuracy and safety of pedicle screw placement in scoliosis associated with Marfan syndrome [16, 17].

## Methods

Patients with scoliosis associated with Marfan syndrome receiving posterior fixation from January 2007 to March 2016 were included in the study. The diagnoses were made by two spinal surgeons and a genetic specialist according to the Ghent nosology. Pre-operative and post-operative CT scans were performed on all patients. The medical records including gender, age at the time of surgery, magnitude of pre and post-operative curves, curve pattern, number of inserted pedicle screws and complications were collected for all patients. The magnitude of the curve was evaluated by using the Cobb method. Pedicle screw placements were performed with the help of fluoroscopy or O-arm navigation system.

### Surgical technique

Free-hand pedicle screw placement technique [20]

Entry point for the thoracic spine was selected as the junction of the outer third and inner two thirds of the superior facet joint. For the lumbar spine, entry point was the junction of the mammillary process, inferior aspect of the transverse process and pars interarticularis. The entry was made with a probe into the pedicle. Pathfinder was used to make further passage. After removing pathfinder, the intact of cortices were checked by a ball-tipped probe. If any perforation was detected, the entry would be remade in an adjusted direction. Fluoroscopy was used to verify the position of each screw and to revise screw position [35].

### O-arm navigation technique

A dynamic reference frame (DRF, tracker) was installed on a spinous process. Then, the first 3D scan was acquired, and images of each section were recorded. The 3D images were reconstructed and then transferred to the navigation station (StealthStation S7 Navigation System; Medtronic, Minneapolis, MN, USA). Under the guiding of navigation, the entry point and trajectory were designed. An awl was used to make the entry and the pedicle tract was made with a probe. By a navigated screwdriver, the pedicle screw was placed to a proper depth. The position and screw trajectory were displayed on the monitor [36] (Fig.1).

### CT Evaluation

Post-operative CT-scanning was performed for all the patients on instrumented segments. The assessment was performed on a PACS image system. An attending surgeon assessed all CT scanning to analyze screw accuracy. Pedicle perforations were classified as medial, lateral or anterior and categorized into one of four grades: ≤ 2 mm as Grade 1, 2.1–4.0 mm as Grade 2, 4.1–6.0 mm as Grade 3, ≥6.1 mm as Grade 4. Fully contained screws or with medial wall perforation ≤ 2 mm or with lateral wall perforation ≤ 6 mm and without injury of visceral organs were considered acceptable, otherwise were unacceptable [35] (Fig.2).

### Statistical analysis

The variables of pedicle screw as defined by fully contained screw accuracy and acceptably positioned screw accuracy were compared using Fisher (two-tailed) exact test as to: 1) convex side or concave side; 2) thoracic spine or lumbar spine; 3) free-hand or O-arm navigation.

## Results

A series of 75 patients were included. There were 35 males and 40 females whose ages ranged from 10 to 28 years old, with an average age of 16.4 years old. The pre-operative Cobb angles were 45° to 165°, with an average angle of 74.8°. The post-operative Cobb angles ranged from 16° to 85°, with an average angle of 30.8°. Free-hand pedicle screw insertion technique was used for 63 patients and O-arm navigation technique for 12 patients.

976 screws were placed, 713 screws (73.1%) were fully contained within the cortical boundaries of the pedicle. 263 (26.2%) screws were in perforation of the pedicle wall. 136 were lateral perforations, 102 medial and 25 anterior. 924 (94.7%) screws were acceptably positioned, and 52 (5.3%) were unacceptably positioned. Of the 263 screws in perforation, 173 were rated as Grade 1, 52 as Grade 2, 32 as Grade 3, and 6 as Grade 4.

783 screws were placed by free-hand technique and 193 by O-arm navigation technique. 241 perforations occurred by free-hand technique and 22 by O-arm navigation technique. The perforation rate was higher using free-hand technique than O-arm navigation technique (30.8% VS. 11.4%, P < 0.05).

In the thoracic spine, 592 screws were placed and 384 in the lumbar spine. The perforation rate was higher in the lumbar region than in the thoracic region (34.1% VS. 22.3%, *P* < 0.05).

507 screws were placed in the convex side of the spine and 469 in the concave side. 106 perforations occurred in the convex side and 157 in the concave side. The perforation rate was higher in the concave side than in the convex side (33.5 VS. 21.9%, *P* < 0.05).

No injury of visceral organs especially aorta erosion was noted in these patients. For free hand group, 6 cases of dural tear caused by misplaced screws occurred, and 4 cases developed cerebro-spinal fluid leak. Drainage and pressure dressings were applied for these patients, and no infection was observed. Leg pain caused by misplaced screws was observed in 7 cases (3 at L1, 2 at L2 and 2 at L3), and 2 cases (1 at L1 and 1 at L2) simultaneously complained of leg weakness. Revision surgery was conducted to remove the misplaced screws for these 2 patients. Conservative treatment was applied for the other 5 patients without leg weakness. All symptoms resolved at final follow-up. For O-arm navigation group, only 1 case of dural tear caused by misplaced screws was observed, and no cerebro-spinal fluid leak for this patient.

## Discussion

Numerous studies have investigated the safety and efficacy of pedicle screw placement in spinal surgery. However, the perforation rates vary from 1% to 43% in literature, and the variation seems to be attributed to the postoperative imaging technique, the grading scheme used, and the threshold for classifying a screw as misplaced [17–19]. CT has been the standard method for postoperative evaluation of pedicle screw placement. Compared to plain radiography, CT shows good sensitivity and reliability with regard to pedicle screw placement in the thoracic and lumbar spine [20, 21]. Previous literature described a 62% negative predictive value for CT [22] and indicated that CT might overestimate the rate of pedicle screw misplacement. However, CT is still the best method to evaluate the accuracy of screw placement [19].

The grading system evaluating perforation with 2 mm increments was utilized in this study. This method is widely considered the standard method to evaluate the accuracy of pedicle screw placement in spine surgery. The system is based on anatomic measurements featuring good reliability and readiness [16, 18]. In addition, we also classified screws as acceptably positioned or unacceptably positioned. With regard to medial wall perforation and safety, one study based on the results of CT myelogram in one patient postulated that up to 4 mm is acceptable in thoracic spine pedicle screw placement because it allows for a safety zone in which there is 2 mm for the epidural space and 2 mm for the CSF with the subarachnoid space [23]. Additionally, the spinal canal in patients with Marfan syndrome is enlarged because of dura ectasia that leads to a larger safe margin between the spinal cord and medial wall of the pedicel, thus a 2 mm threshold is sufficient to ensure an intact spinal cord. With regard to lateral perforation, 6 mm is considered acceptable, which was confirmed by a cadaveric study indicating 6.8 mm as acceptable [24]. Assessment was also made with regard to impingement of visceral organs especially devastating aorta erosion.

Belmont et al. [16] reported a 99% screw accuracy in thoracic spine. Smorgick [25] reported an 87.5% rate of fully contained screws in 25 patients. Lehman [26] evaluated the accuracy of pedicle screws placement in pediatric patients, and reported a 10.5% rate of wall perforation. In this study, the rate of perforation is 26.8% and the rate of acceptably positioned screws is 96.7%. Both the accuracy and perforation rate in this cohort were higher than those in idiopathic scoliosis and trauma cases, which could be explained by the following factors: first, the curves in this cohort were larger leading to bigger rotation of vertebrae. It would pose more difficulties for screw placement [27]; second, patients with Marfan syndrome have thinner pedicles compared with patients with idiopathic scoliosis or trauma, which increase the possibility of pedicle perforation [35].

Unlike AIS, the perforations occurred more frequently in lumbar spine than in thoracic spine (57/193 VS. 24/102). Double major curves and lumbar curves were more frequently seen in Marfan syndrome, and pedicles of lumbar spines were always extremely thin; thus, even when pedicle screws were placed in the right directions, perforations would inevitably occur (Fig.3). The perforation rate is higher in the concave side than the convex side which can be explained by the smaller pedicles in the concave side of a scoliotic spine [28].

Many methods to enhance safety and accuracy of pedicle screw placement have been developed, such as funnel technique, intraoperative C-arm image intensifier, CT-assisted technique and computer-assisted technique [29–31]. With these techniques, the safety and accuracy of pedicle screw placement can be improved significantly [32]. However, some of these techniques need special equipment and the operative time may be prolonged. Moreover, prolonged exposure to irradiation could cause additional harm to either patients or surgeons. Previously, C-arm fluoroscopy was used to obtain anatomic orientation for screw placement. With the introduction of O-arm based navigation systems, we have achieved improved accuracy with screw placement.

There is a significant learning curve associated with pedicle screw placement [34], and all screws in this study were placed by experienced surgeons.

Placement-related complications include neurologic impingement, aorta injury, dural tear, vertebral fracture [14, 15, 17, 33]. Suk et al. [17] reported one case with transitory paraparesis due to medial perforation of the pedicle causing delayed epidural hematoma. After removal of screw and decompressive laminectomy the paraparesis resolved. Silverstre [14] reviewed a cohort of scoliotic patients undergoing a posterior approach and found screw related complications of pedicle fracture, pleural effusion, injury to the spinal cord, nerve root and aorta. Papin et al. [33] report a case with symptoms of epigastric pain, right foot tremor at rest and abnormal sensation in the legs caused by 2 screws at T8 and T10 that penetrated medially by 4 mm. After screw revision surgery, and complete recovery occurred 1 month later. In this series, no impingement of visceral organs occurred. 7 cases of dural tear caused by misplaced screws occurred, and 4 cases developed cerebro-spinal fluid leak. Drainage and pressure dressings were applied for these patients, and no infection was observed. Leg pain was observed in 7 cases, and 2 cases simultaneously complained of leg weakness. Revision surgery was conducted to remove the misplaced screws for these 2 patients. The 2 screws were both placed in the concave side of lumbar spine, and the pedicles were extremely thin. We suggest that it is better to skip these thin pedicles and increase the screw density in neighboring levels instead. Conservative treatment was applied for the other 5 patients without leg weakness. All the symptoms were finally relieved.

## Conclusions

In conclusion, placement of pedicle screw in Marfan syndrome is accuracy and safe. O-arm navigation was an effective modality to ensure the safety and accuracy of screw placement. Special attention should be paid when screws were placed at the lumber spine and the concave side of spine deformity to avoid the higher rate of complications.

## Abbreviations

MFS: Marfan syndrome.
